# Supplementary material for: Optimizing deep learning-based segmentation of densely packed cells using cell surface markers
Source: BMC Med Inform Decis Mak. 2024 May 15;24:124. doi: 10.1186/s12911-024-02502-6 (PMC11094866; doi:10.1186/s12911-024-02502-6)
Supplement: Supplementary file 1 — Additional file 1. Contains additional figures supporting the study results. [file 12911_2024_2502_MOESM1_ESM.pdf]

Optimizing Deep Learning-Based Segmentation of Densely Packed Cells using Cell Surface Markers

Sunwoo Han, Khamsone Phasouk, Jia Zhu, Youyi Fong

A Supporting figures

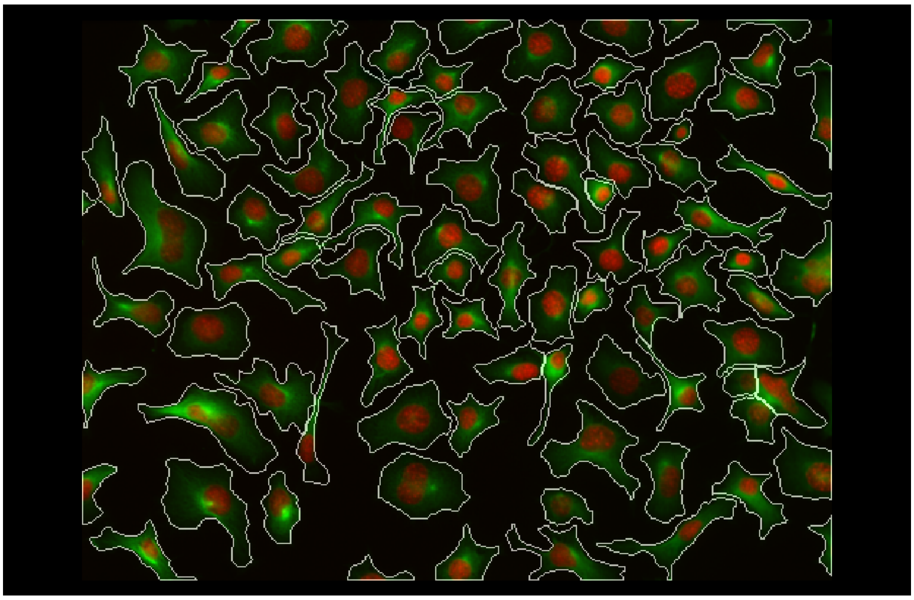

Figure A.1: Cellpose training image 1, size 512×383. Red is the nucleus channel, green is the cytoplasm channel, cell mask is shown in white.

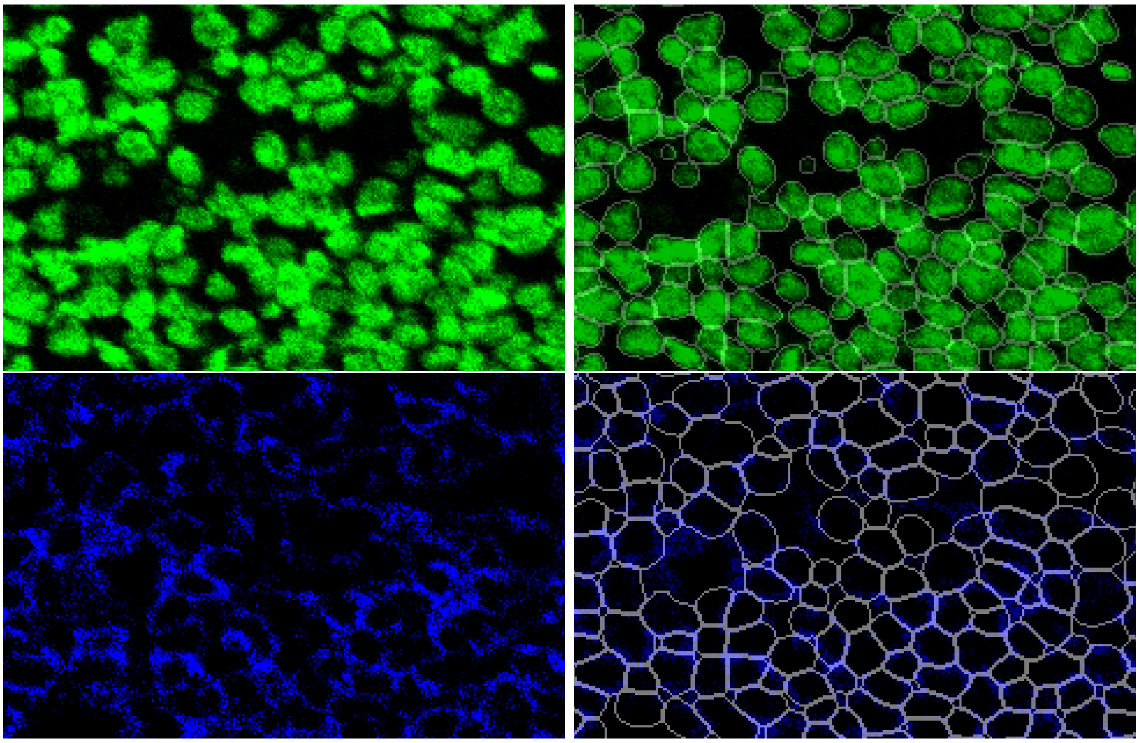

Figure A.2: TissueNet training image 1. A randomly selected patch of size 241×157 is shown. Top left: nuclear channel; top right: nuclear channel + nuclear masks; bottom left: cytoplasm channel; bottom right: cytoplasm channel + cytoplasm masks.

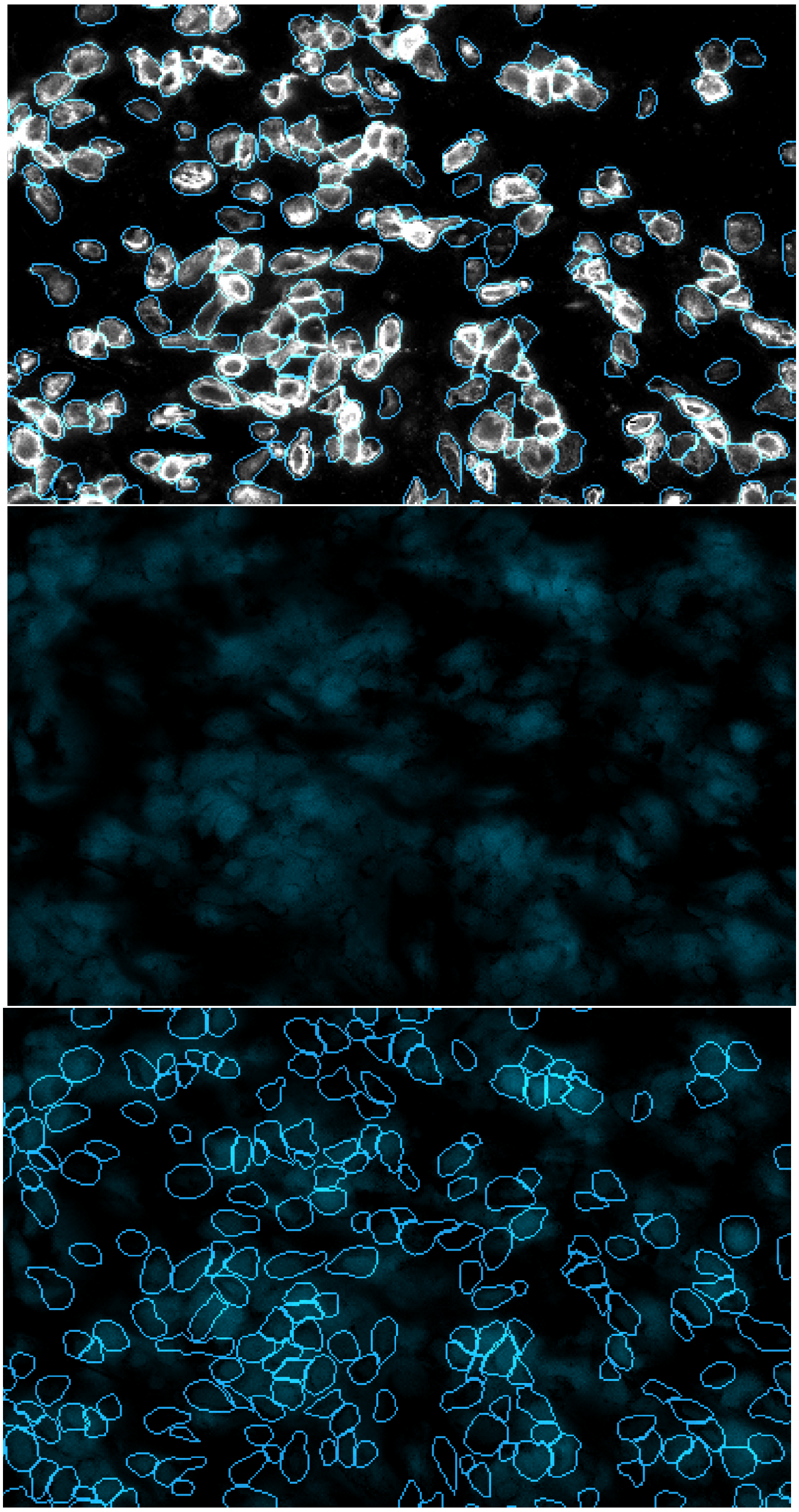

Figure A.3: A randomly selected patch of size 398×252 from 2_CD3 training image. Top: CD3 image with masks; middle: DAPI staining, bottom: DAPI staining + CD3 masks.

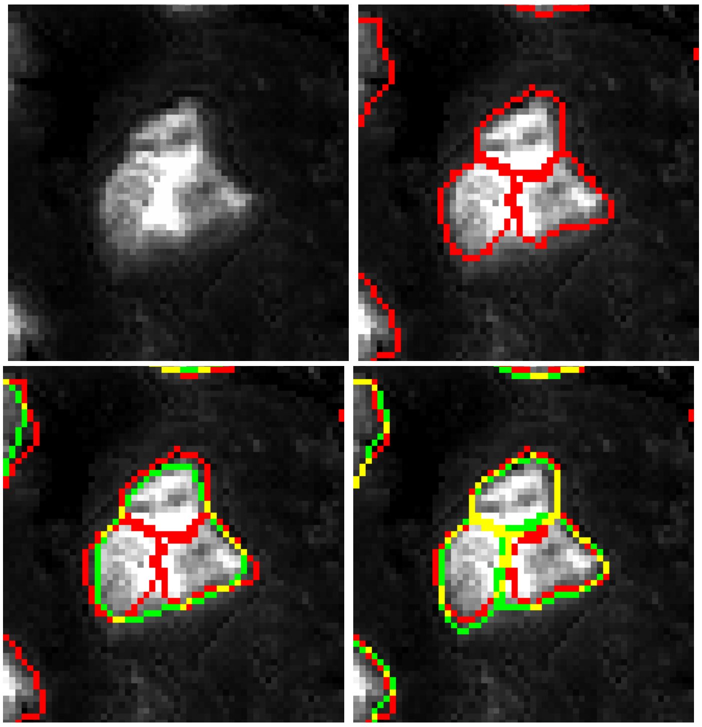

Figure A.4: A portion of the 5_CD3 test image. Top left: CD3 intensities. Top right: ground truth masks in red. Bottom left: ground truth masks in red and *cyto* masks in green. Bottom right: ground truth masks in red and *cyto-train7* masks in green.

with rotation	without rotation
0.692	0.722
0.700	0.701
0.703	0.710
0.702	0.720
0.707	0.707
0.707	0.710

Table A.1: mAP of Cellpose models from six replicates with different random seeds.

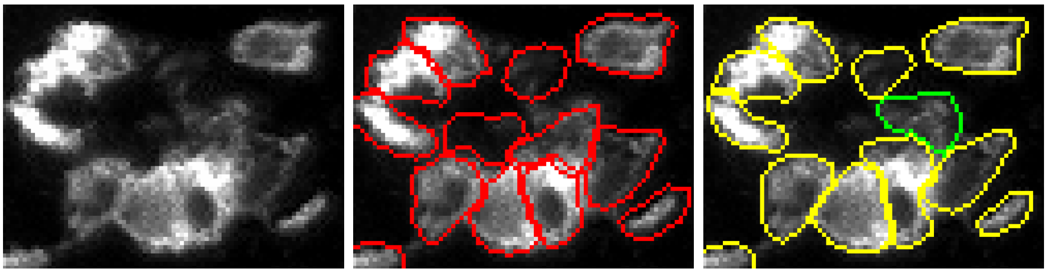

Figure A.5: A portion of 2_CD3 test image. Left: CD3 intensities. Middle: ground truth masks (the first round of expert-drawn masks) in red. Right: the second round of expert-drawn masks, yellow masks are true positives and green masks are false positives using the ground truth masks as reference (IoU threshold 0.5).

B Generalizability of the fine-tuned models

Table B.1 shows the average precision (AP) of seven fine-tuned models starting from cyto on each of the seven test strips. The last row shows the mAP, which corresponds to the first row in Table 3 of the main text. A number highlighted in blue is an 'out-of-sample' test result in the sense that no part of the image that is tested on has been used in training. For example, in the 7_CD3 row, the first seven test results are all blue because only Train7 was trained on an imageset including a portion of 7_CD3.

For ease of interpretation, we plot the highlighted APs in Figure B.1 below. The mAPs are also plotted as a reference. As the figure shows, the 'out-of-sample' performance mostly follows the same trend as the mAPs, including a dip at Train1, a sharp increase at Train2, and gradual but continued improvements afterwards, with a single exception for the 6_CD3 test strip at Train3.

	cyto	Train1	Train2	Train3	Train4	Train5	Train6	Train7
1_CD8	0.605	0.574	0.644	0.656	0.666	0.676	0.713	0.703
2_CD3	0.570	0.493	0.681	0.709	0.727	0.724	0.731	0.749
3_CD4	0.679	0.536	0.701	0.764	0.778	0.789	0.782	0.762
4_CD3	0.478	0.397	0.561	0.604	0.608	0.622	0.625	0.628
5_CD3	0.409	0.391	0.550	0.594	0.625	0.655	0.679	0.690
6_CD3	0.480	0.429	0.605	0.580	0.633	0.640	0.685	0.686
7_CD3	0.408	0.348	0.506	0.522	0.561	0.568	0.623	0.639
mAP	0.518	0.453	0.607	0.633	0.657	0.668	0.691	0.694

Table B.1: APs of the fine-tuned models on individual test strips. Starting from *cyto*, the models Train1 to Train7 adds one new training image at a time in the same order as the rows are ordered. The numbers highlighted in blue are 'out-of-sample' test values.

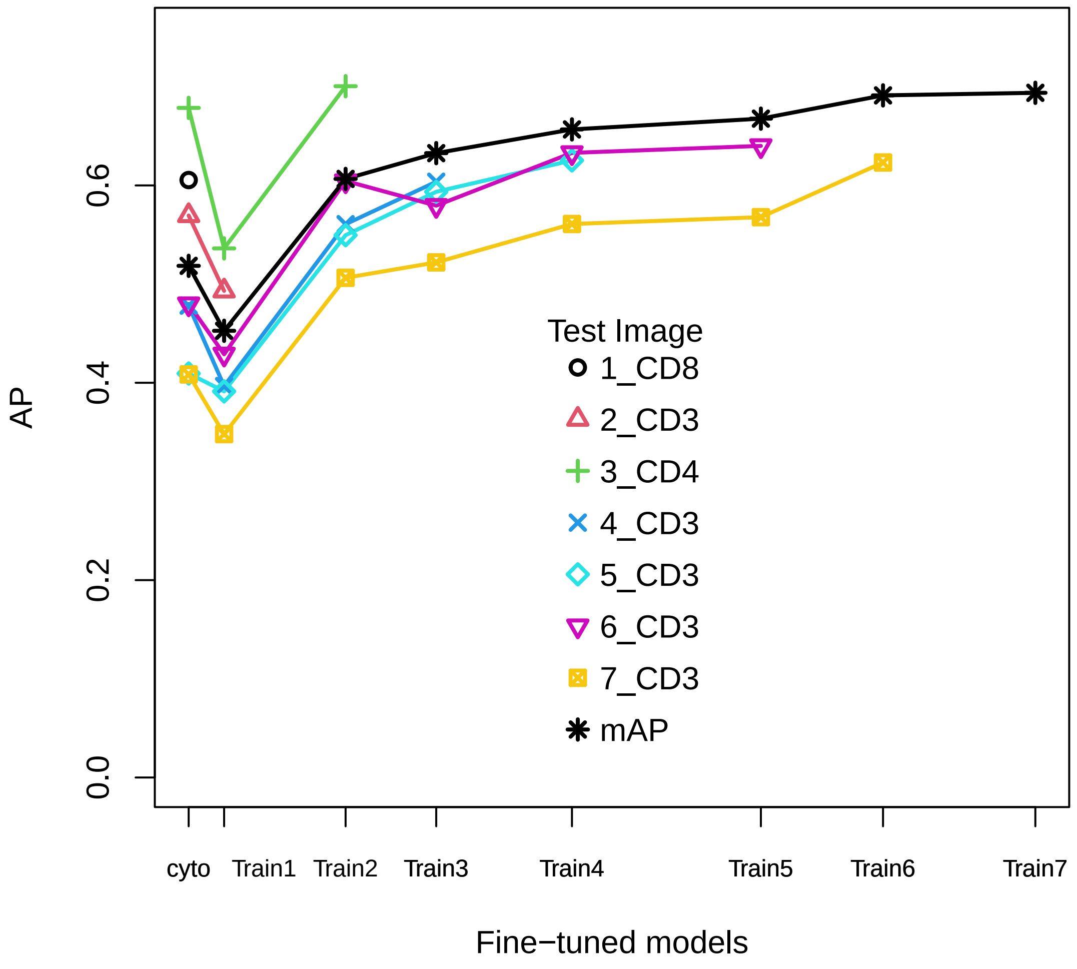

Figure B.1: APs of the fine-tuned models on individual test strips. Starting from *cyto*, the models Train1 to Train7 adds one new training image at a time in the order shown in the figure legend. Only the 'out-of-sample' test values are shown.

As a second way to address the concern of generalizability, we worked on a new image, 8_CD3, no part of which was used for training. The following figure shows two 112×112 patches from the image. For the patch in the first row, the AP is .421 between gt and cyto and 0.639 between gt and cyto-train7; for the patch in the second row, the AP is 0.333 between gt and cyto and 0.593 between gt and cyto-train7.

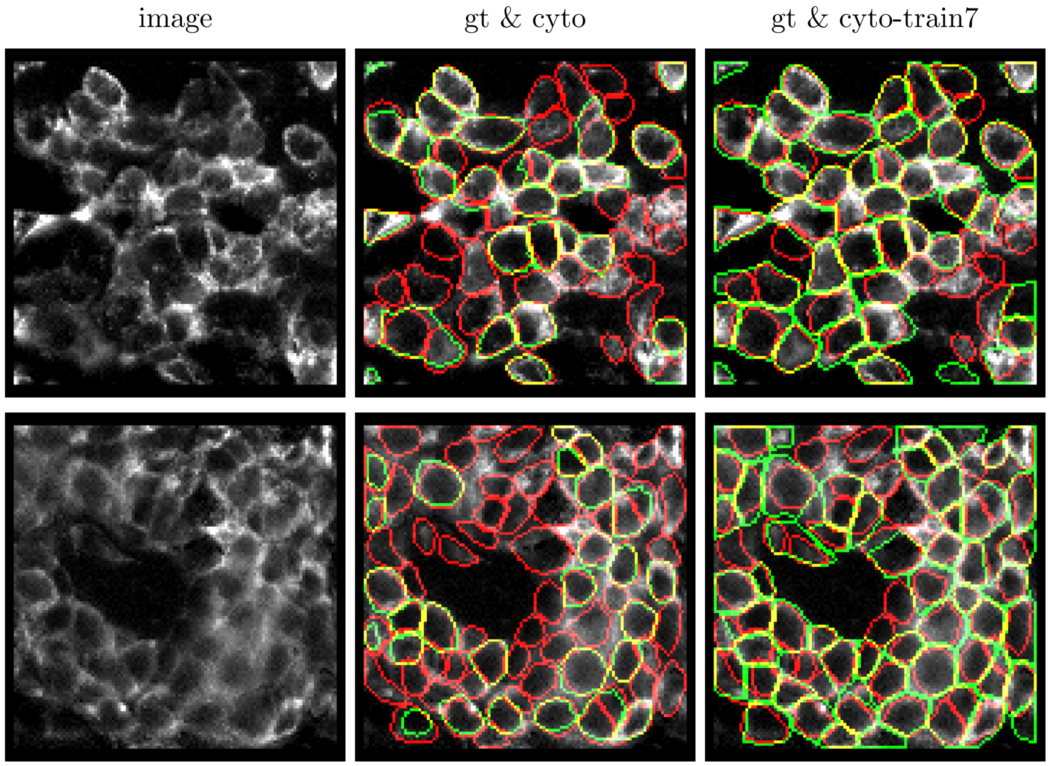

Figure B.2: Predictions on two patches from a new image, 8_CD3. The first column displays immunostaining using an anti-CD3 antibody, while the second and third columns present the image overlaid with masks. gt: ground truth, cyto: Cellpose cyto, cyto-train7: fine-tuned model. Ground truth masks are shown in red. Predicted masks are shown in green.

In summary, our imageset contains ∼10,000 training instances collected over several months from seven images. There is variation in both image quality and annotation style among the seven images. To study optimization and comparison of several cell segmentation models, we performed the training/testing split by stratifying on images. The analyses in this section show that the fine-tuned models perform well on test data from images that they have not seen before.

Finally, we performed a 7-fold cross validation to test the generalization capability question. Specifically, we trained the model with 6 images and tested on the remaining image and repeated the procedure 7 times. The results are summarized in the Table B.2.

	cyto	cv
1_CD8	0.504	0.709
2_CD3	0.379	0.617
3_CD4	0.559	0.588
4_CD3	0.539	0.698
5_CD3	0.635	0.696
6_CD3	0.409	0.639
7_CD3	0.540	0.727
mAP	0.509	0.668

Table B.2: Each row shows, for one test image, the AP of the Cellpose cyto model and the AP of a model fine-tuned with the other six images. The last row shows the average AP from each column.
